# Hybrid atrial fibrillation ablation in patients with persistent atrial fibrillation or failed catheter ablation

**DOI:** 10.1007/s12471-019-1228-3

**Published:** 2019-02-04

**Authors:** M. I. H. Al-Jazairi, M. Rienstra, T. J. Klinkenberg, M. A. Mariani, I. C. Van Gelder, Y. Blaauw

**Affiliations:** 1 Department of Cardiology, University of Groningen, University Medical Center Groningen, Groningen, Groningen, The Netherlands; 2 Department of Cardiothoracic Surgery, University of Groningen, University Medical Center Groningen, Groningen, The Netherlands

**Keywords:** Atrial fibrillation, Hybrid, Ablation, Thoracoscopic, Pulmonary vein isolation, Single-stage

## Abstract

**Background:**

Combined ‘hybrid’ thoracoscopic and percutaneous atrial fibrillation (AF) ablation is a strategy used to treat AF in patients with therapy-resistant symptomatic AF. We aimed to study efficacy and safety of single-stage hybrid AF ablation in patients with symptomatic persistent AF, or paroxysmal AF with failed endocardial ablation, and assess determinants of success and quality of life.

**Methods:**

We included consecutive patients undergoing single-stage hybrid AF ablation. First, we performed epicardial ablation, via thoracoscopic access, to isolate the pulmonary veins and superior caval vein and to create a posterior left atrial box. Thereafter, isolation was assessed endocardially and complementary endocardial ablation was performed, followed by cavotricuspid isthmus ablation. Efficacy was assessed by 12-lead electrocardiography and 72-hour Holter monitoring after 3, 6 and 12 months. Recurrence was defined as AF/atrial flutter/tachycardia recorded by electrocardiography or Holter monitoring lasting >30 s during 1‑year follow-up.

**Results:**

Fifty patients were included, 57 ± 9 years, 38 (76%) men, 5 (10%) paroxysmal, 34 (68%) persistent and 11 (22%) long-standing persistent AF. At 1‑year 38 (76%) maintained sinus rhythm off antiarrhythmic drugs. Majority of recurrences were atrial flutter (9/12 patients). Success was associated with type of AF (*p* = 0.039). Patients with paroxysmal AF had highest success, patients with longstanding persistent AF had lowest success. Seven (14%) patients had procedure-related complications. Quality of life improved after ablation in patients who maintained sinus rhythm.

**Conclusion:**

Success of single-stage hybrid AF ablation was 76% off antiarrhythmic drugs, being associated with type of AF. Quality of life improved significantly, Procedure-related complications occurred in 14%.

## What’s new?

- Single-stage hybrid ablation is highly effective in patients with paroxysmal AF after prior failed endocardial ablations.
- Type of AF is the main determinant of single-stage hybrid ablation success.
- Hybrid AF ablation led to improvement in patients’ quality of life.

## Introduction

At present there is only limited evidence that a rhythm control strategy for atrial fibrillation (AF) improves patients‘ mortality and morbidity [1–4]. However, many patients are moderately to severely symptomatic, necessitating a rhythm control strategy [5].

Antiarrhythmic drugs (AADs), often being used as first-line rhythm control strategy [5], are only moderately successful in maintaining sinus rhythm (SR) during long-term follow-up [6]. Endocardial pulmonary vein isolation (PVI) has now become an established, effective and safe therapy for patients with paroxysmal AF [5, 7]. However, in patients with persistent and long-standing persistent AF, PVI alone is less effective, even after multiple procedures [8], or with the inclusion of additional endocardial ablation lines or targets [9]. This is likely due to advanced electro-anatomic remodelling of the atria and the extra-pulmonary vein triggers being more prominent [10].

The surgical Cox-Maze procedure showed higher success in patients with persistent and long-standing persistent AF [11]. It is, however, a complex and invasive procedure. Hybrid AF ablation combines surgical thoracoscopic epicardial atrial ablation with endocardial atrial catheter ablation. The potential benefit of this combined approach is that incomplete epicardial ablation lines can be completed in the same session, via an endocardial approach. In contrast to the Cox-Maze procedure, thoracoscopic epicardial ablation is less invasive. At present, there is still little data about the outcome of single-stage hybrid AF ablation with few centres reporting conflicting results [12, 13]. Our aim is to investigate the efficacy and safety of hybrid AF ablation at our centre, to explore factors determining its success, and to assess its effect on the quality of life.

## Methods

### Patient population

The Hybrid AF Ablation study, Clinicaltrials.gov registration number NCT02516033, is a prospective, single-centre, observational study performed at the University Medical Center Groningen, the Netherlands. A total of 50 consecutive patients were included between January 2015 and June 2016. The institutional review board approved the study protocol, and all patients provided written informed consent. Patients with symptomatic persistent or long-standing persistent AF, or paroxysmal AF with two or more failed catheter ablations were candidates for this procedure. Patients with a history of cardiac surgery, and patients with a body mass index (BMI) of 35 or over were excluded.

### Definitions

AF was defined as paroxysmal (all AF episodes lasting less than 7 consecutive days), persistent (at least some AF episodes lasting more than 7 consecutive days, but could still spend periods in SR), or long-standing persistent AF (AF episode lasting more than 365 consecutive days) [5].

### Study design

At baseline, we assessed clinical history, physical examination, current medication, an electrocardiogram, blood samples, 24-hour Holter monitoring, echocardiography, cardiac computed tomography scan and quality of life.

Prior to the procedure, a trans-oesophageal echocardiogram was performed to exclude the presence of a thrombus in the left atrium and atrial appendage. Outpatient clinic visits with electrocardiography (ECG) recording were scheduled at 1, 3, 6 and 12 months after procedure. At 3, 6 and 12 months, a 72-hour Holter monitoring was performed. At 12 months, quality of life was re-assessed. During all visits we collected information about AF, atrial flutter (AFL) or atrial tachycardia (AT) recurrences documented by the general practitioner, during emergency room visits or during hospital admissions, as well as adverse events associated with the procedure. AADs were continued during the first 3 months, and then discontinued in symptom-free patients. All patients were on acenocoumarol treatment for at least 4 weeks before the procedure. Periprocedurally, acenocoumarol was not interrupted (target international normalised ratio (INR) 2–2.5) and continued thereafter for at least 3 months.

### Hybrid AF ablation procedure

The procedure was performed under general anaesthesia. A double lumen endotracheal tube was placed for selective lung ventilation.

### Thoracoscopic procedure

Three thoracoscopic ports were placed on the right side, one 5‑mm camera port at the fifth intercostal space (midaxillary) and two working ports in a diamond shape, 5‑mm and 12-mm, respectively. Afterwards, the pericardium was opened anterior to the phrenic nerve. The transverse and oblique sinuses were opened with blunt dissection. A track light dissector (Lumitip, AtriCure) was then used to guide a rubber band and position the ablation device (Isolator Synergy Clamps, AtriCure) around the right pulmonary veins (PVs). We ablated the right PVs and then performed ablation of the superior caval vein, using the same bipolar clamp. Ablation for the roof and inferior line of the posterior left atrial box was performed using a linear ablation device (Coolrail, AtriCure). We tested isolation of the right PVs and the superior caval vein epicardially with a 4-polar catheter (Supreme, St. Jude Medical), with additional ablation if necessary. The left PVs were ablated via a similar left thoracoscopic approach. To complete the posterior box, the roof and inferior line were also made from the left side meeting the ablation lines made from the right side. The isolation of the ablated structures (including the box) was tested again. In patients with previous catheter PV ablation, we tested PV isolation epicardially with the 4‑polar Supreme catheter before ablation, but performed epicardial ablation irrespective of whether the PVs were isolated or not. Finally, in patients with CHA_2_DS_2_-VASc (congestive heart failure, hypertension, age ≥75 [doubled], diabetes mellitus, prior stroke [doubled]-vascular disease, age 65–74, sex category) scores of 2 or higher, we performed surgical exclusion of the left atrial appendage (LAA), using a minimally invasive occlusion device (AtriClip, AtriCure). LAA exclusion did not involve any ablation and was performed exclusively for stroke reduction. Oral anticoagulants were resumed after the procedure in all patients according to their CHA_2_DS_2_-VASc score, regardless of undergoing LAA exclusion [5].

### Percutaneous procedure

**Fig. 1:**
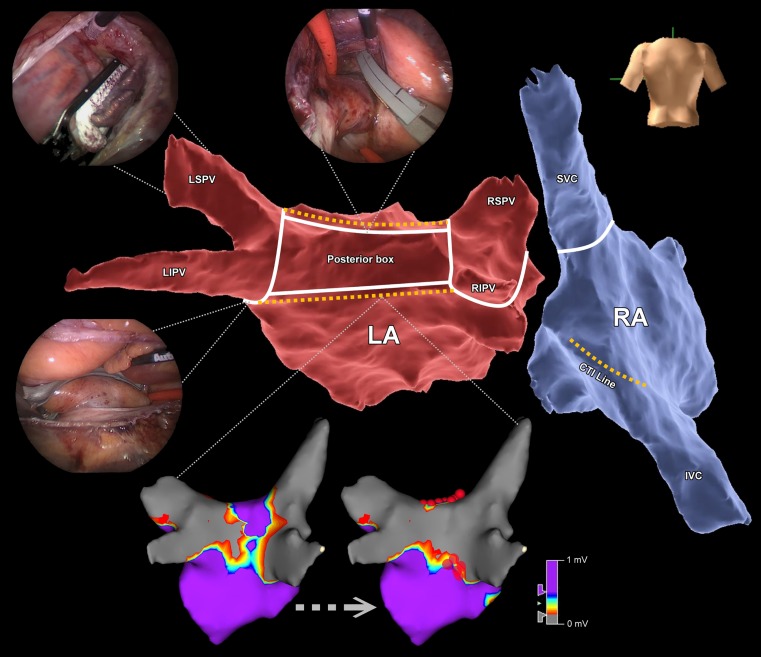
The standard set of ablation lines during the hybrid AF ablation procedure (*White lines* epicardial lines, *yellow dotted lines* endocardial lines; *AF* atrial fibrillation*, LA* left atrium, *RA* right atrium, *LSPV* left superior pulmonary vein, *LIPV* left inferior pulmonary vein, *RSPV* right superior pulmonary vein, *RIPV* right inferior pulmonary vein, *CTI* cavotricuspid isthmus, *SVC* superior caval vein, *IVC* inferior caval vein)

After the surgical procedure, access to the left atrium was established via the femoral vein with a single trans-septal puncture using an 8.5F sheath (Agilis steerable sheath, St. Jude Medical), after which 100E/kg bolus dose of unfractionated heparin was given (target activating clotting time >300 s). Three dimensional electroanatomical mapping was performed using NavX Ensite Velocity (NavX, St. Jude Medical). Electroanatomical maps of the left atrium were created with a circular multipolar catheter (Inquiry optima or Inquiry AFocusII, St. Jude Medical). We performed ablation with an open irrigated 4 mm tip contact force ablation catheter (TactiCath Quartz CF ablation catheter, St. Jude Medical). We performed additional endocardial ablation if the veins were not isolated, surgical lines were incomplete or AF/AFL/AT persisted,. In case of AF, complex fractionated atrial electrogram (CFAE) ablation was performed in the left atrium and within the coronary sinus. CFAE was defined as low voltage electrograms having a very short cycle length or fractionation composed of multiple (more than 2) deflections or perturbation of the baseline with continuous deflection of a prolonged activation complex. CFAE detection was done by visual inspection; automated software was not employed. Endpoint of CFAE ablation was conversion of AF to AT/AFL or SR. If AF persisted despite extensive ablation, we performed cardioversion. In case of AT/AFL the arrhythmia was mapped and ablated. Additionally, in all patients, the cavotricuspid isthmus (CTI) line was ablated, or tested for bidirectional block if the patient underwent CTI ablation in the past. Finally, re-induction of AF was attempted using rapid pacing (with cycle lengths as short as 180 ms) in the coronary sinus. Upon induction of AF, we stopped pacing and monitored the duration of AF/AFL/AT. Additional mapping and ablation was performed if sustained AF/AFL/AT was triggered and lasted long enough to allow mapping it (Fig. 1).

### Efficacy and safety endpoints

The primary endpoint was the first AF/AFL/AT recurrence documented by ECG, Holter monitoring (episode lasting more than 30 s), or an event recorder during 12-month follow-up, excluding recurrences occurring in the first 90 days (blanking period) [14]. The secondary endpoints were safety and patient’s quality of life. Safety was defined as the absence of any complications as described by the 2017 expert consensus statement on catheter and surgical ablation of AF [15], quality of life assessment was assessed using the European Heart Rhythm Association (EHRA) score, the Toronto AF Severity Scale (AFSS) and Short Form 36 questionnaires (SF-36).

### Statistical analysis

Patient and procedural characteristics are given as mean ± standard deviation or median and interquartile range for continuous variables and as number of patients with percentages for categorical variables. Kaplan-Meier plot and univariate cox-regression analysis were used to show and compare outcomes. Analysis was done using R package (version 3.4.3), and a *p*-value smaller than 0.05 was considered statistically significant.

## Results

### Clinical characteristics

Tab. 1 shows the baseline clinical characteristics. Mean age was 57 ± 9 years, 38 (76%) patients were men, five (10%) had paroxysmal, 34 (68%) persistent and 11 (22%) long-standing persistent AF. Time since first AF diagnosis was 5.1 (2.1–8.4) years and 25 (50%) patients had prior failed catheter ablation(s).

**Table 1:** Patient clinical characteristics of study population at baseline (*n* = 50)

age (years)	57 ± 9
total history of AF (years)	5.1 (2.1–8.4)
men	38 (76%)
*type of AF*	
paroxysmal	5 (10%)
persistent	34 (68%)
long-standing	11 (22%)
previous failed catheter ablation(s)	25 (50%)
previous CTI ablation	10 (20%)
heart failure	2 (4%)
hypertension	23 (46%)
diabetes mellitus	4 (8%)
coronary artery disease	2 (4%)
stroke or TIA	3 (6%)
CHA_2_-DS_2_-VASc score	1 (0–2)
*physical examination*	
systolic blood pressure (mm Hg)	129 ± 14
diastolic blood pressure (mm Hg)	82 ± 10
BMI (kg/m^2^)	28.0 ± 3.8
obesity (BMI > 30)	12 (24%)
*echocardiography*	
interventricular septum thickness (mm)	10.6 ± 1.9
LA volume index (ml/m^2^)	40 ± 11
LVEF (%)	55 ± 6
use of antiarrhythmic drugs	19 (38%)

*AF* atrial fibrillation, *CHA*_*2*_*-DS*_*2*_*-* congestive heart failure, hypertension, age ≥ 75 years [doubled], diabetes mellitus, prior stroke [doubled]-vascular disease age 65–74, sex category,* CTI* cavotricuspid isthmus, *TIA* transient ischaemic attack, *BM*I body mass index, *LA* left atrial, *LVEF* left ventricular ejection fraction

### Procedural data

**Fig. 2:**
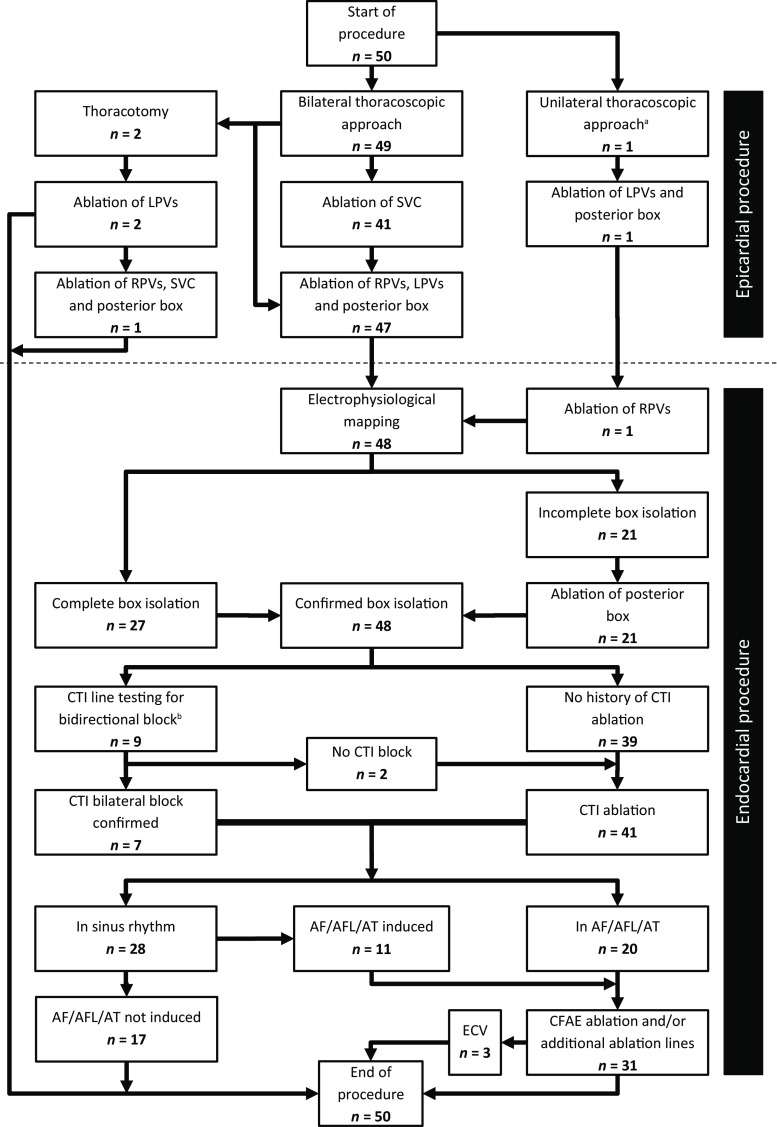
Flow chart showing the steps followed during the procedure for the patients in the study cohort. ^a^The surgeon couldn’t gain access to the left atrium from the right side because of local adhesions. ^b^In patients with previous catheter CTI ablation. (*LPV* left pulmonary vein*, SVC* superior caval vein, *RPV* right pulmonary vein,* CTI* cavotricuspid isthmus,* AF/AFL/AT* atrial fibrillation/atrial flutter/atrial tachycardia,* ECV* electrical cardioversion, *CFAE* complex fractionated atrial electrogram)

Tab. 2 and Fig. 2 show the procedural data. Two (4%) patients did not undergo the endocardial procedure due to bleeding during epicardial ablation necessitating thoracotomy. Additionally, six patients had a pacemaker or an implantable cardioverter defibrillator and therefore superior caval vein ablation was not performed to avoid damaging the leads. In all patients, all PVs were isolated following surgical ablation. In 27 (54%) patients, endocardial mapping demonstrated posterior box isolation after the epicardial procedure. In 21 (42%) patients, additional endocardial ablation resulted in complete box isolation. In 7 (14%) of these 21 patients only the superior line was incomplete after the epicardial procedure, in 3 (6%) patients only the inferior line was incomplete, and in 11 (22%) patients gaps were found in both the superior and inferior lines. In 2 (4%) patients endocardial mapping was not performed due to a bleeding complication resulting in thoracotomy (see Fig. 2).

**Table 2:** Procedural data

	all patients (*n* = 50)
*rhythm at start of procedure*	
– SR	22 (44%)
– AF	28 (56%)
total procedure time (minutes)	396 ± 45
*surgical part*	
– patient preparation (minutes)	55 ± 12
– surgical procedure (minutes)	218 ± 47
– percutaneous part (minutes)	128 ± 36
total number of epicardial applications	85 ± 27
– right pulmonary veins	8 ± 2
– left pulmonary veins	8 ± 3
– superior line	28 ± 13
– inferior line	34 ± 16
– superior caval vein	2 ± 1
patients undergoing endocardial ablation:	48 (96%)
– to complete box isolation	21 (42%)
– for CTI line	41 (82%)
– for additional lines (to stop AFL/AT)	19 (38%)
– for CFAE ablation (to stop AF)	22 (44%)
confirmed box isolation after epicardial ablation^a^	27 (54%)
LAA closure	15 (30%)

*CTI* cavotricuspid isthmus, *SR* sinus rhythm, *AF* atrial fibrillation, *AFL* atrial flutter, *AT* atrial tachycardia, *CFAE* complex fractionated atrial electrogram, *LAA* left atrial appendage

^a^ through endocardial electrophysiological mapping

### Efficacy

At 1‑year follow-up, 38 (76%) patients maintained SR following hybrid AF ablation. Recurrent atrial arrhythmias occurred in 12 (24%) patients. The majority of these recurrences (9 patients) were atypical AFL, with the remaining 3 patients having AF recurrences. Seven of these nine patients with AFL recurrences underwent endocardial re-ablation, six of them completed at least 6 months of follow-up and all six maintained SR until their last follow-up. The seventh patient had to undergo a second endocardial re-ablation due to quick recurrence of AFL. Overall, only two (4%) patients used AADs at 1 year, both had earlier AF/AFL recurrences (Tab. 3).

**Table 3:** Results after 1‑year follow-up. Success at 1‑year follow-up

	all patients (*n* = 50)
sinus rhythm maintenance^a^	38 (76%)
total recurrences	12 (24%)
– atrial fibrillation recurrences	3 (6%)
– atrial flutter recurrences	9 (18%)
antiarrhythmic drug use at 1 year	2 (4%)
endocardial re-ablation	7 (14%)
electrical or chemical cardioversion	9 (18%)

^a^ Off antiarrhythmic drugs and without re-ablation

**Table 4:** Results after 1‑year follow-up. List of the major procedural complications

	all patients (*n* = 50)	recovered without sequelae
total major complications	7 (14%)	4 (8%)
– bleeding requiring thoracotomy	2 (4%)	2 (4%)
– permanent phrenic nerve paralysis	2 (4%)	0 (0%)
– pericardial and pleural effusion	1 (2%)	1 (2%)
– pleural effusion	1 (2%)	1 (2%)
– pacemaker implantation	1 (2%)	0 (0%)
mortality	0 (0%)	–

### Safety

Seven (14%) patients suffered complications during and after the procedure. Two (4%) had bleeding during the epicardial procedure necessitating thoracotomy; one due to an injury to the pulmonary artery and the other due to injury to the right inferior pulmonary vein. Both patients recovered completely without any sequelae. Two (4%) had permanent phrenic nerve injury, 1 (2%) developed pericardial and pleural effusion requiring drainage, 1 (2%) pleural effusion requiring drainage, and 1 (2%) was known with a latent sick sinus syndrome before the procedure, requiring a DDD-pacemaker after restoration of SR. Total hospital stay was 6 ± 2 days (Tab. 4).

### Determinants of success

Success of hybrid AF ablation showed a significant association with type of AF at baseline (100% in paroxysmal AF versus 79% in persistent AF versus 55% in long-standing persistent AF, *p* = 0.039; Fig. 3a and b) and history of previous failed catheter ablation (90% versus 62% for patients without previous catheter ablation, *p* = 0.020; Fig. 3c).

**Fig. 3:**
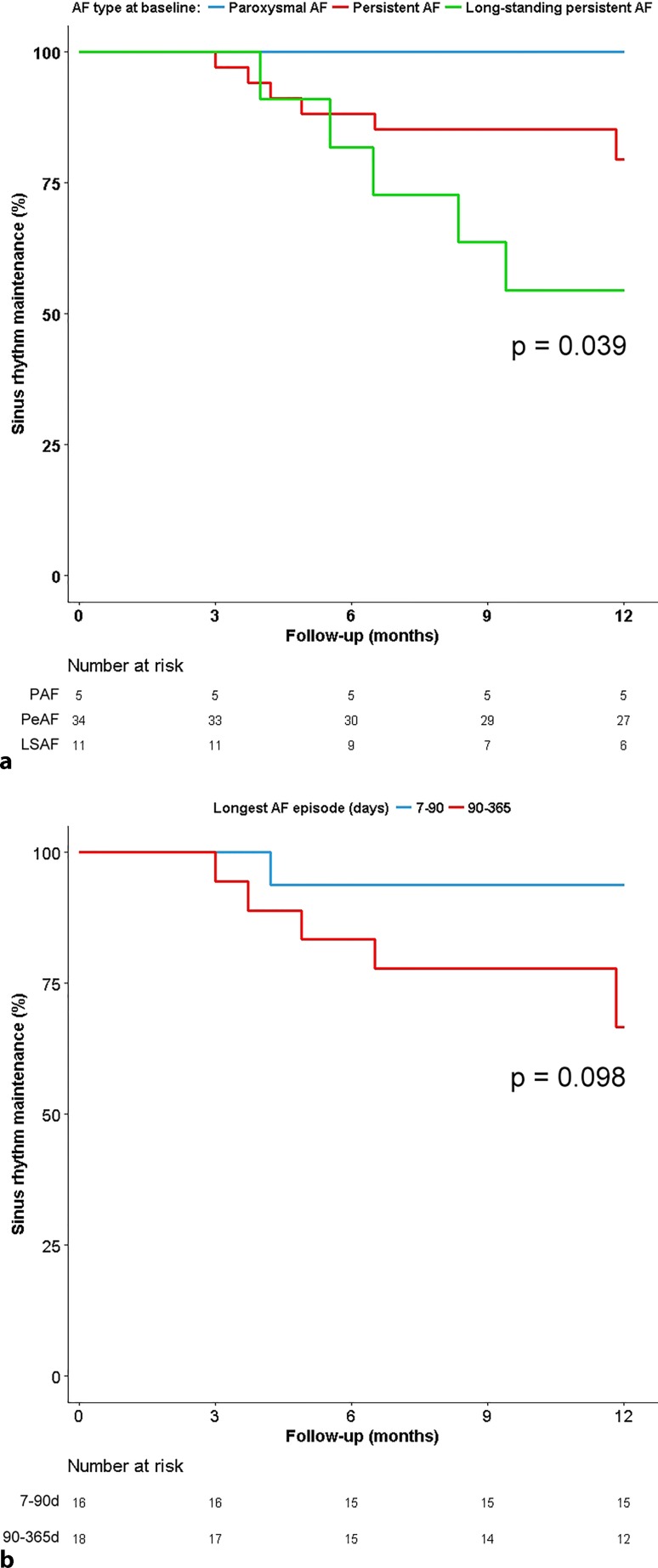
Kaplan-Meier plot showing outcome of the procedure. Outcome according to: **a** type of AF at baseline. **b** duration of longest AF episode in patients with persistent AF. **c** history of catheter ablation. *AF* atrial fibrillation. *AF* atrial fibrillation

**Fig. 3:**
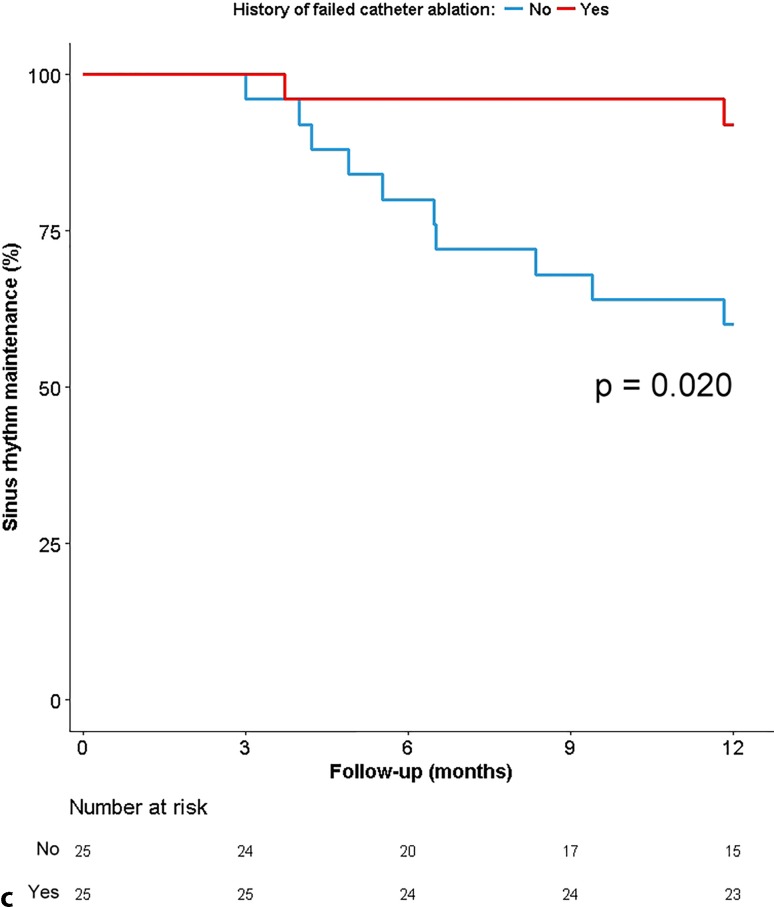
(Continued)

### Quality of life

The EHRA score (Fig. 4a), the AFSS questionnaire (Fig. 4b) and the SF-36 questionnaire (Fig. 4c) at 1‑year follow-up all showed significant improvement compared with baseline, predominantly in patients who maintained SR.

**Fig. 4:**
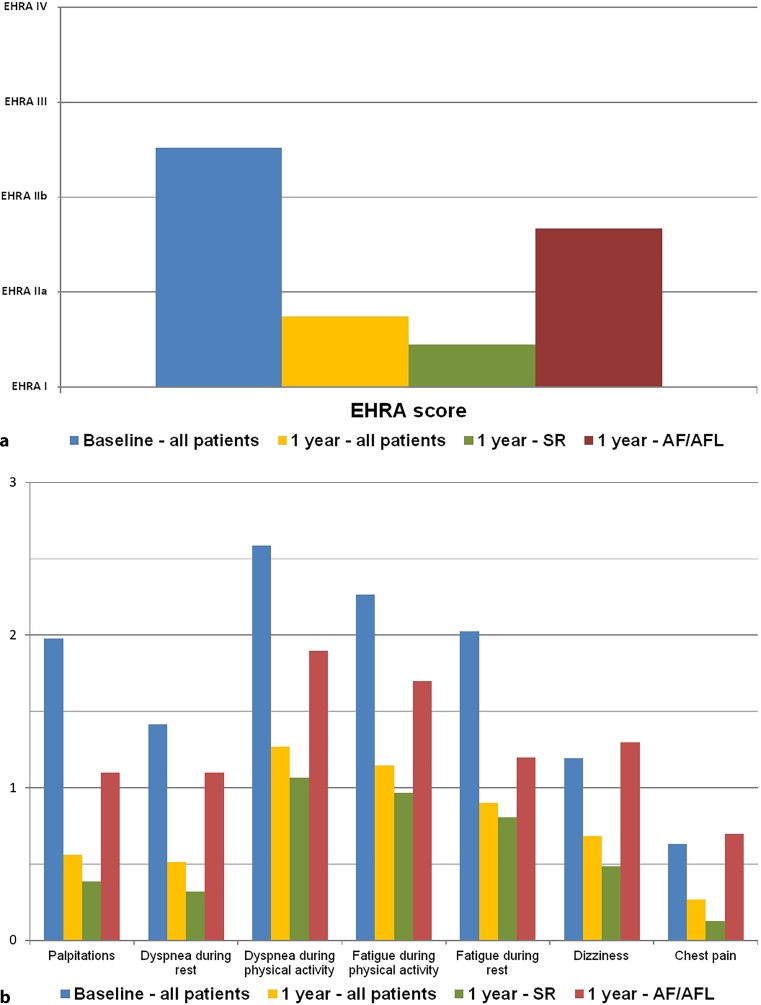
Scores and results at baseline and 1 year. **a** Average EHRA score at baseline and 1 year. **b** Results of AF Severity Scale questionnaire at baseline and 1 year. (*EHRA* European Heart Rhythm Association, *AF* atrial fibrillation)

**Fig. 4:**
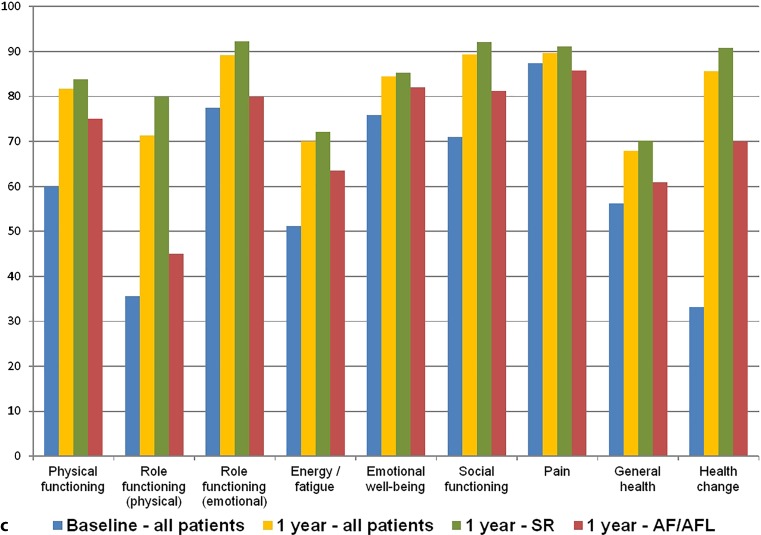
(Continued) **c** Results of Short Form 36 questionnaire at baseline and 1 year. (*EHRA* European Heart Rhythm Association, *AF* atrial fibrillation)

## Discussion

Our study showed that in patients with previous failed catheter ablation or persistent AF, single-stage hybrid AF ablation was successful in 76% of patients off AADs at 1‑year follow-up. Success was significantly lower in patients with long-standing persistent AF. Endocardial touch-up was needed in 42% of patients to achieve posterior box isolation. We have included posterior box isolation as an endpoint for the procedure, because this area is more likely to harbour AF triggers or drivers, especially in patients with persistent AF [16], or contain substrate for re-entry, because of its proximity to the pulmonary veins and its complex fibre orientation [17]. Therefore, isolating the posterior box may contribute to a better outcome by eliminating these AF drivers and reducing the area of the atrial tissue capable of sustaining AF. Additionally, all variants of Maze-like procedures have posterior box isolation as a part of their protocol [18].

Two earlier studies reported on single-stage hybrid AF ablation in 50 patients or more. Pison et al. [12] described 74% success off AADs in 78 patients and Gehi et al. [19] reported a 66% success rate in 101 patients, including patients with concomitant AAD therapy. Our study population was comparable with the former study, but was younger and had less co-morbidities than the latter.

Our complication rate was higher than both Pison et al. (8%) and Gehi et al. (6%) [12, 19] reported in hybrid ablation but lower than Boersma et al. in the FAST trial (23%) for minimally invasive surgical AF ablation [20]. Important to note here that Gehi et al. reported two deaths, while no mortality was observed in our cohort. Additionally, our complication rate was higher than the 12% rate reported in a recent contemporary endocardial ablation trial (Fire and ICE) [21]. However, multiple endocardial re-ablations may also increase the cumulative complication risk for endocardial ablation.

In our analysis, type of AF was significantly associated with success, i. e. more persistent forms of AF were associated with poorer outcome. Our finding are not unexpected since long-standing persistent AF is associated with more extensive structural atrial changes, driven by associated comorbidities and AF itself [22]. This renders the ablation lines insufficient to abolish AF due to the presence of larger atrial area capable of sustaining AF [23]. In line with this, we observed a trend towards a better outcome in patients with a shorter episode of persistent AF. Of interest, our analysis also showed that in patients with one or more previous failed catheter ablations, including both paroxysmal and persistent AF patients, hybrid AF ablation resulted in excellent 1‑year arrhythmia-free follow-up. The success rate for longstanding persistent AF was moderate but higher than reported in endocardial ablation studies [8, 9]. Of interest, the majority of these recurrences were AFL episodes. Endocardial re-ablation was highly effective, although the follow-up period was relatively short.

Finally, quality of life assessment showed significant improvement at 1‑year follow-up, especially in the physical parameters. This improvement was, as expected, more pronounced in patients who maintained SR during the follow-up period.

### Limitations

The number of patients in this analysis is still too small to draw definitive conclusions. Additionally, due to the lack of continuous heart rhythm monitoring during the follow-up period, it is possible that we failed to document some asymptomatic recurrences of atrial arrhythmias.

## Conclusion

Hybrid AF ablation is an effective treatment for persistent AF or paroxysmal AF after failed catheter ablation, but the risk of procedure-related complications should be taken into consideration. Therefore, accurate patient selection is of utmost importance, weighing the risks of complications against the potential gain in the patient’s quality of life and the chance of success. Patients with paroxysmal AF after failed catheter ablation, or persistent AF with relatively shorter AF episodes appear to be the best candidates.
